# Association of lipid profiles with severity and outcome of acute ischemic stroke in patients with and without chronic kidney disease

**DOI:** 10.1007/s10072-020-04791-x

**Published:** 2020-10-13

**Authors:** Ailing Zhang, Wenjing Deng, Bin Zhang, Mengyang Ren, Long Tian, Jihui Ge, Jinjuan Bai, Hao Hu, Ling Cui

**Affiliations:** 1 Department of Neurology, People’s Hospital of Zhengzhou, Zhengzhou City, 450003 Henan Province China; 2 The Neurology Intensive Care Unit, The First Affiliated Hospital of Zhengzhou University, Zhengzhou City, 450000 Henan Province China

**Keywords:** Ischemic stroke, Chronic kidney disease, Lipoproteins, Severity

## Abstract

**Background:**

Contribution of lipid profiles to stroke severity and outcome was inconclusive, whether chronic kidney disease (CKD) (estimated glomerular filtration rate < 60 mL/min/1.73 m^2^) affects the association has not been investigated. We aim to evaluate this relationship.

**Methods:**

A retrospective study of consecutive acute ischemic stroke patients was performed. We assessed the risk of severe stroke with the National Institutes of Health Stroke Scale (NIHSS) ≥ 5 at admission and poor outcome with the modified Rankin Scale (mRS) ≥ 3 at discharge. Multivariate stepwise logistic regression models were adopted to study interaction and independent association of lipid components with stroke severity and outcome according to lipid level quartiles by CKD stratification.

**Results:**

Among the 875 included patients (mean age 64.9 years, 67.8% males), 213 (24.3%) presented with CKD. Elevated low-density lipoprotein cholesterol (LDL-C) was independently associated with severe stroke in patients with CKD (*P* for trend = 0.033) than in those without CKD (*P* for trend = 0.121). The association between the level of LDL-C and stroke severity was appreciably modified by CKD (*P*_interaction_ = 0.013). Compared with without CKD patients in the lowest LDL-C quartile, the multivariable-adjusted risk of severe stroke increased significantly by 2.9-fold (95% CI 1.48–5.74) in patients with CKD in the highest LDL-C quartile. No significant association was observed between lipid components and early outcome in patients with and without CKD.

**Conclusion:**

LDL-C levels are positively associated with stroke severity in only patients with CKD, with an interactive impact of LDL-C and CKD on ischemic stroke in the acute phase.

## Introduction

Stroke and renal dysfunction have rapid growth trends in many countries with increasing prevalence rates of hypertension, diabetes mellitus, hyperlipidemia, and obesity [1]. The prevalence of chronic kidney disease (CKD) is estimated to be about 8–16% in the general population, and it is most common among cardiovascular disease and stroke populations [2]. Furthermore, the impact of CKD on stroke seems to be greater in Asian patients than non-Asian patients [2]. Patients with CKD often have dyslipidemia [3, 4], which is an important pathogenic factor of atherosclerosis and a modifiable risk factor for cardio- and cerebrovascular disease. Renal impairment affects blood lipid metabolism; on the other hand, dyslipidemia increases the risk of CKD [5, 6]. There was a significant effect of the interaction between lipid dyslipidemia and renal impairment.

Previous studies indicated that some lipid components were associated with increased severity of neurological injury and adverse outcomes of acute ischemic stroke (AIS) in the general population [7–11]. However, these studies did not stratify analysis differences between patients with and without CKD. Whether the association of lipid components with initial stroke severity and functional outcome could be modified by CKD because of a potential interaction between lipids and low estimated glomerular filtration rate (eGFR) need to be explored further. To optimize stroke management, especially in high-risk AIS populations with CKD, there is a pressing need to further assess whether serum lipid therapeutic targets in CKD patients differ from those in the general population.

The aim of this study was to investigate the association of different lipid levels with severity and functional outcome of AIS patients with and without CKD and evaluate the interaction of lipid profiles and CKD on stroke, including whether the impacts are independent of stroke etiology and other known prognosticators.

## Methods

### Patients

This study was a retrospective observational study of AIS patients who were consecutively admitted to the neurology department of Zhengzhou People’s Hospital (a grade A tertiary hospital) from January 2016 to May 2019. The inclusion criteria were as follows: (1) age ≥ 18 years; (2) acute ischemic stroke was confirmed by computed tomography or magnetic resonance imaging within 48 h after onset. The exclusion criteria were as follows: (1) intracerebral hemorrhage; (2) transient ischemic attack; (3) other etiologies and undetermined stroke; (4) subarachnoid hemorrhage; (5) brain tumors; (6) those treated with intravenous thrombolytic therapy before onset; (7) a modified Rankin Scale (mRS) score > 1 before onset; (8) patients with incomplete clinical data. Patients were given routine treatment for stroke with antiplatelet and anticoagulation according to etiology after admission. All data were evaluated by blindness in retrospective analysis.

Patients baseline data, including demographics, vascular risk factors (diabetes mellitus, ischemic heart disease, hypertension, atrial fibrillation, heart failure, prior stroke, smoking), blood pressure, imaging tests, lipids profile, other laboratory tests, stroke subtypes, initial stroke severity, and functional outcome, were collected and clinically evaluated. Based on clinical information, neuroimaging findings, and laboratory examinations, AIS subtypes were determined according to the Trial of Org 10,172 in Acute Stroke Treatment (TOAST) classification [12].

### Biochemistry

Blood samples were taken after overnight fasting within the first 24 h of hospital admission. A TBA-FX8 biochemistry analyzer (Toshiba, Japan) was used during this study. Routine laboratory analyses, including total cholesterol (TC), triglyceride (TG), high-density lipoprotein cholesterol (HDL-C), low-density lipoprotein cholesterol (LDL-C), homocysteine (HCY), hemoglobin A1c (HbA1c), and serum creatinine, were performed in all enrolled patients.

### Renal function measurement

Assessment of renal function was based on estimated glomerular filtration rate (eGFR) calculated using creatinine measured on the first day after admission with the Chronic Kidney Disease Epidemiology Collaboration (CKD-EPI) equation for Asians [13]. CKD was defined as an eGFR < 60 mL/min/1.73 m^2^.

### Clinical assessment

Initial stroke severity was assessed using the National Institutes of Health Stroke Scale (NIHSS) score at admission. Severe stroke was defined as NIHSS ≥ 5. Functional outcome was evaluated by the modified Rankin Scale (mRS) score at discharge. A poor outcome was defined as mRS ≥ 3 (dependency/death).

### Statistical analysis

Continuous variables were described as means ± standard deviations or medians with interquartile range (IQR). Categorical variables were presented as proportions. Student’s *t* or Mann-Whitney *U* tests were used for continuous variables, while *χ*^2^ or Fisher’s exact tests were used for categorical variables. Baseline lipid variables were further stratified into quartiles, with the lowest quartile of lipids as the reference group. We used multivariate stepwise logistic regression analysis to evaluate the association between different lipid levels and severity and outcomes of stroke by CKD stratification. The effects of interactions were examined in the models with interaction terms. Two-tailed *P* values < 0.05 were considered statistically significant. All analyses were performed using SPSS 25.0 (IBM, USA).

## Results

### Baseline characteristics

A total of 875 patients were included in the present study (mean age (64.9 ± 12.8) years, 67.8% males). Severe stroke occurred in 49.6% (434/875) of patients, poor outcomes were observed in 31.8% of patients (278/875), and 24.3% (213/875) of patients presented with CKD. Clinical characteristics are presented in detail according to CKD status in Table 1. Patients with CKD were older; were more likely to be women; smoked less; had higher systolic blood pressure levels, HCY levels, and baseline NIHSS scores; and higher rates of hypertension, ischemic heart disease, prior stroke, atrial fibrillation, and heart failure than those without CKD. Lipid profiles were not significantly different between the two groups.

**Table 1:** Clinical characteristics of the AIS patients with and without CKD

Variables	All (*n* = 875)	CKD (−) (*n* = 662)	CKD (+) (*n* = 213)	*P* value
Demographics				
Age, year, mean ± SD	64.85 ± 12.79	62.03 ± 12.23	73.63 ± 10.26	0.000
Male, *n* (%)	593 (67.8)	477 (72.1)	116 (54.5)	0.000
BMI, mean ± SD, kg/m^2^	25.26 ± 3.47	25.30 ± 3.43	25.16 ± 3.59	0.615
Smoking (current/past, *n*%)	435 (49.7)	353 (53.3)	82 (38.5)	0.000
Medical history, *n* (%)				
Hypertension	680 (77.7)	497 (75.1)	183 (85.9)	0.001
Diabetes mellitus	361 (41.3)	271 (40.9)	90 (42.3)	0.734
Ischemic heart disease	217 (24.8)	136 (20.5)	81 (38.0)	0.000
Prior stroke	308 (35.2)	215 (32.5)	93 (43.7)	0.003
Atrial fibrillation	97 (11.1)	48 (7.3)	49 (23.0)	0.000
Heart failure	66 (7.5)	35 (5.3)	31 (14.6)	0.000
Clinical characteristics				
SBP, mean ± SD, mmHg	147.50 ± 20.53	145.45 ± 20.02	153.86 ± 20.83	0.000
DBP, mean ± SD, mmHg	84.50 ± 12.78	84.49 ± 12.74	84.52 ± 12.93	0.976
NIHSS at admission (IQR)	4 (2–8)	4 (2–7)	5 (3–9)	0.001
NIHSS at admission < 5	441 (50.4)	354 (53.5)	87 (40.8)	0.001
NIHSS at admission ≥ 5	434 (49.6)	308 (46.5)	126 (59.2)	
MRS < 3	597 (68.2)	479 (72.4)	118 (55.4)	0.000
MRS ≥ 3	278 (31.8)	183 (27.6)	95 (44.6)	
Stroke subtypes				
Large-artery atherosclerosis	488 (55.8)	360 (54.4)	128 (60.1)	0.000
Cardioembolism	125 (14.3)	83 (12.5)	42 (19.7)	
Small-vessel occlusion	262 (29.9)	219 (33.1)	43 (20.2)	
Laboratory characteristics				
TG, median (IQR),mmol/L	1.37 (0.99–1.96)	1.42 (1.01–1.99)	1.27 (0.94–1.83)	0.072
TC, mmol/L	4.41 ± 1.06	4.38 ± 1.04	4.48 ± 1.10	0.264
HDL, mmol/L	1.04 ± 0.28	1.03 ± 0.26	1.07 ± 0.32	0.060
LDL, mmol/L (mmol/L)	2.66 ± 0.85	2.64 ± 0.83	2.71 ± 0.91	0.298
Hcy, median (IQR), μmol/L	14.38 (11.22–18.17)	13.85 (11.00–17.61)	15.72 (12.64–19.38)	0.000
HbA1C,%	6.96 ± 1.43	7.01 ± 1.49	6.80 ± 1.22	0.074
eGFR, mL/min/1.73 m^2^	71.10 (60.03–83.99)	76.23 (68.51–88.20)	51.12 (39.83–56.35)	0.000

*CKD*, chronic kidney disease; *BMI*, body mass index; *SBP*, systolic blood pressure; *DBP*, diastolic blood pressure; *NIHSS*, National Institute of Health stroke scale; *mRS*, modified Rankin Scale; *TG*, triglyceride; *TC*, total cholesterol; *HDL-C*, high-density lipoprotein cholesterol; *LDL-C*, low-density lipoprotein cholesterol; *Hcy*, homocysteine; *HbA1c*, hemoglobin A1c; *eGFR*, estimated glomerular filtration rate

### Association between lipid levels and the severity of stroke by CKD status

Table 2 shows the results of the multivariate logistic regression analysis of severe stroke according to lipid level quartiles in patients with and without CKD. The age- and sex-adjusted ORs of stroke severity increased with increasing LDL-C quartiles in the groups with and without CKD (both *P* for trend< 0.05). A significant trend was observed in only those with CKD (*P* for trend = 0.033) and not in those without CKD (*P* for trend = 0.121) after the multivariate adjustment for relevant confounding factors. However, there was no significant association between other lipid variables and stroke severity regardless of CKD status (all *P*_trend_> 0.05).

**Table 2:** Adjusted odds ratios (ORs) for severe stroke according to lipid level quartiles in patients with and without CKD (NIHSS ≥ 5)

Lipid variables	Quartile 1 (Reference)	Quartile 2 OR (95% CI)	Quartile 3 OR (95% CI)	Quartile 4 OR (95% CI)	*P* for trend
TC (mmol/L)	TC < 3.61	3.61 ≤ TC < 4.34	4.34 ≤ TC < 5.04	TC ≥ 5.04	
CKD (−)					
Model 1	1.000	0.812 (0.525–1.257)	1.449 (0.927–2.266)	1.304 (0.836–2.034)	0.047
Model 2	1.000	0.858 (0.548–1.341)	1.379 (0.871–2.183)	1.244 (0.777–1.991)	0.175
CKD (+)					
Model 1	1.000	0.809 (0.349–1.877)	1.418 (0.650–3.093)	2.140 (0.915–5.001)	0.151
Model 2	1.000	0.683 (0.280–1.669)	1.166 (0.512–2.658)	1.721 (0.699–4.233)	0.272
TG (mmol/L)	TG < 0.99	0.99 ≤ TG < 1.37	1.37 ≤ TG < 1.96	TG ≥ 1.96	
CKD (−)					
Model 1	1.000	0.788 (0.501–1.240)	0.939 (0.604–1.460)	0.820 (0.522–1.287)	0.695
Model 2	1.000	0.764 (0.478–1.220)	0.905 (0.574–1.428)	0.772 (0.482–1.239)	0.616
CKD (+)					
Model 1	1.000	0.597 (0.287–1.244)	0.703 (0.309–1.598)	0.962 (0.407–2.275)	0.478
Model 2	1.000	0.553 (0.253–1.207)	0.681 (0.281–1.649)	1.046 (0.406–2.697)	0.356
HDL-C (mmol/L)	HDL-C < 0.85	0.85 ≤ HDL-C < 0.99	0.99 ≤ HDL-C < 1.19	HDL-C ≥ 1.19	
CKD (−)					
Model 1	1.000	0.854 (0.550–1.327)	0.586 (0.376–0.914)	0.857 (0.544–1.349)	0.106
Model 2	1.000	0.857 (0.544–1.352)	0.591 (0.374–0.932)	0.927 (0.575–1.493)	0.102
CKD (+)					
Model 1	1.000	0.722 (0.313–1.667)	0.954 (0.412–2.211)	0.611 (0.266–1.404)	0.562
Model 2	1.000	0.781 (0.321–1.900)	0.868 (0.358–2.105)	0.592 (0.243–1.443)	0.668
LDL-C (mmol/L)	LDL-C < 2.08	2.08 ≤ LDL-C < 2.58	2.58 ≤ LDL-C < 3.10	LDL-C ≥ 3.10	
CKD (−)					
Model 1	1.000	1.093 (0.703–1.699)	1.625 (1.044–2.527)	1.694 (1.087–2.642)	0.036
Model 2	1.000	1.115 (0.710–1.749)	1.557 (0.986–2.459)	1.588 (0.994–2.536)	0.121
CKD (+)					
Model 1	1.000	1.503 (0.675–3.347)	2.534 (1.097–5.852)	4.153 (1.770–9.746)	0.007
Model 2	1.000	1.441 (0.621–3.345)	2.471 (1.025–5.958)	3.553 (1.438–8.776)	0.033

*CKD*, chronic kidney disease; *TG*, triglyceride; *TC*, total cholesterol; *HDL-C*, high-density lipoprotein cholesterol; *LDL-C*, low-density lipoprotein cholesterol; *95% CI*, 95% confidence interval

Model 1: adjusted for age and sex

Model 2: as for model 1 plus body mass index, systolic blood pressure, diastolic blood pressure, smoking, hypertension, diabetes mellitus, ischemic heart disease, prior stroke, atrial fibrillation, heart failure, homocysteine, hemoglobin A1c

**Fig. 1:**
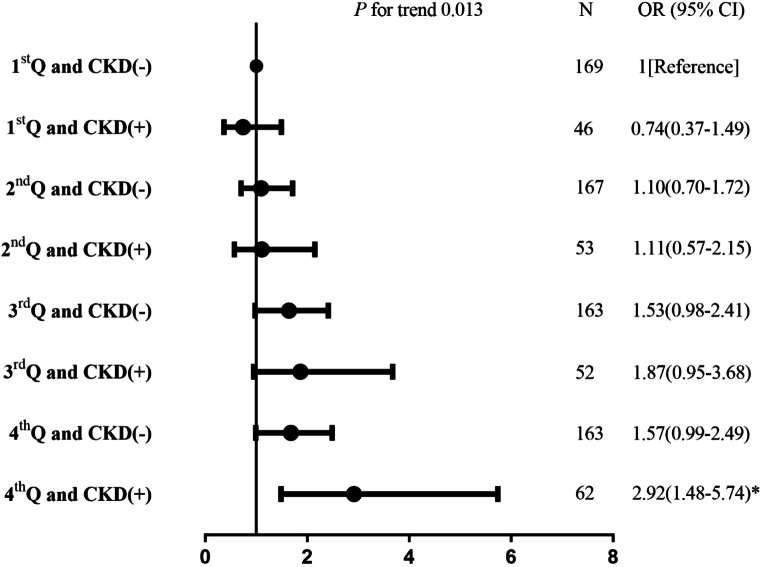
The effect of the interaction of LDL-C quartile levels and CKD on the severity of stroke. Adjusted for age, sex, body mass index, systolic blood pressure, diastolic blood pressure, smoking, hypertension, diabetes mellitus, ischemic heart disease, prior stroke, atrial fibrillation, heart failure, homocysteine, hemoglobin A1c. *P = 0.002 (vs 1st Q and CKD (−))

The effect of the interaction of LDL-C quartile levels and CKD on the severity of stroke is shown in Fig. 1. The association between the levels of LDL-C and stroke severity was appreciably modified by CKD status (*P*_interaction_ = 0.013). Compared with non-CKD subjects in the lowest LDL-C quartile, the multivariable-adjusted risk of severe stroke increased significantly by 2.9-fold (95% CI: 1.48–5.74) in subjects with CKD in the highest LDL-C quartile.

### Association between lipid levels and the outcome of stroke by CKD status

Table 3 shows the results of the logistic regression analysis of poor outcome according to lipid level quartiles in patients with and without CKD. With elevated LDL-C levels, the age- and sex-adjusted ORs of a poor outcome increased gradually in the groups without CKD (*P* for trend = 0.033), while HDL-C presented an inverse correlation trend in the groups with and without CKD (both *P* for trend< 0.05), but the significant differences disappeared after adjustment for relevant confounding factors (all *P* for trend> 0.05).

**Table 3:** Adjusted odds ratios (ORs) for poor outcome of stroke according to lipid level quartiles in patients with and without CKD (mRS ≥ 3)

Lipid variables	Quartile 1 (Reference)	Quartile 2 OR (95% CI)	Quartile 3 OR (95% CI)	Quartile 4 OR (95% CI)	*P* for trend
TC (mmol/L)	TC < 3.61	3.61 ≤ TC < 4.34	4.34 ≤ TC < 5.04	TC ≥ 5.04	
CKD (−)					
Model 1	1.000	0.734 (0.442–1.220)	1.142 (0.695–1.877)	1.265 (0.770–2.078)	0.184
Model 2	1.000	0.626 (0.298–1.316)	0.563 (0.274–1.159)	0.612 (0.284–1.318)	0.393
CKD (+)					
Model 1	1.000	1.231 (0.521–2.911)	1.360 (0.621–2.979)	1.600 (0.693–3.695)	0.742
Model 2	1.000	1.491 (0.381–5.832)	1.179 (0.329–4.220)	1.452 (0.380–5.554)	0.925
TG (mmol/L)	TG < 0.99	0.99 ≤ TG < 1.37	1.37 ≤ TG < 1.96	TG ≥ 1.96	
CKD (−)					
Model 1	1.000	0.820 (0.495–1.360)	0.869 (0.530–1.424)	0.968 (0.583–1.608)	0.855
Model 2	1.000	0.841 (0.396–1.790)	0.840 (0.399–1.768)	0.932 (0.431–2.015)	0.959
CKD (+)					
Model 1	1.000	1.070 (0.521–2.196)	0.770 (0.340–1.745)	0.815 (0.350–1.902)	0.841
Model 2	1.000	2.383 (0.674–8.427)	2.134 (0.566–8.051)	1.180 (0.285–4.891)	0.478
HDL-C (mmol/L)	HDL-C < 0.85	0.85 ≤ HDL-C < 0.99	0.99 ≤ HDL-C < 1.19	HDL-C ≥ 1.19	
CKD (−)					
Model 1	1.000	0.882 (0.545–1.426)	0.453 (0.271–0.756)	0.486 (0.288–0.820)	0.003
Model 2	1.000	1.518 (0.742–3.104)	0.662 (0.304–1.446)	0.750 (0.341–1.654)	0.138
CKD (+)					
Model 1	1.000	0.368 (0.154–0.876)	0.330 (0.139–0.779)	0.306 (0.129–0.727)	0.033
Model 2	1.000	0.383 (0.095–1.551)	0.214 (0.054–0.847)	0.268 (0.063–1.129)	0.158
LDL-C (mmol/L)	LDL-C < 2.08	2.08 ≤ LDL-C < 2.58	2.58 ≤ LDL-C < 3.10	LDL-C ≥ 3.10	
CKD (−)					
Model 1	1.000	0.902 (0.536–1.517)	1.336 (0.807–2.210)	1.783 (1.086–2.928)	0.033
Model 2	1.000	0.581 (0.278–1.213)	0.524 (0.246–1.116)	0.834 (0.390–1.784)	0.292
CKD (+)					
Model 1	1.000	0.668 (0.290–1.539)	1.604 (0.702–3.666)	1.719 (0.762–3.876)	0.084
Model 2	1.000	0.287 (0.075–1.101)	0.728 (0.209–2.540)	0.662 (0.177–2.471)	0.312

*CKD*, chronic kidney disease; *TG*, triglyceride; *TC*, total cholesterol; *HDL-C*, high-density lipoprotein cholesterol; *LDL-C*, low-density lipoprotein cholesterol; *95% CI*, 95% confidence interval

Model 1: adjusted for age and sex

Model 2: as for model 1 plus body mass index, systolic blood pressure, diastolic blood pressure, smoking, hypertension, diabetes mellitus, ischemic heart disease, prior stroke, atrial fibrillation, heart failure, homocysteine, hemoglobin A1c, baseline NIHSS score, and stroke subtype

## Discussion

In this study, we found that with elevated LDL-C levels, the severity of stroke increased. We demonstrated that only increased LDL-C levels were independently related to more severe neurological deficit in AIS patients with CKD than in those without CKD, implying that the association between LDL-C levels and stroke severity was modified by kidney function status. Data regarding the relationship between lipid levels and stroke severity have been controversial [14–16]. Some studies reported that low HDL-C was associated with the severity of stroke in general population [15, 16], another study argued that this association only existed in young adult stroke patients [14]. Whereas a meta-analysis showed that TG levels were linked to the severity of stroke [8]. These studies did not consider eGFR decline as an important confounder or adjusted for it in only the multivariable analysis without stratification. Whether the effects of lipid profiles on stroke in patients with CKD differ from the effect on the general population remains unclear. We founded that compared with non-CKD subjects in the lowest quartile of LDL-C, the multivariable-adjusted risk of severe stroke increased significantly by 2.9-fold (95% CI: 1.48–5.74) in subjects with CKD in the highest LDL-C quartile. There may be a significant interaction of elevated LDL-C levels and low eGFR on initial stroke severity.

Dyslipidemia exists in the early stages of CKD, aggravating renal impairment [6, 17, 18]. Lipid-modifying drugs could delay deterioration of kidney function [19, 20]. Reduced kidney function affected lipoprotein metabolism, that is, changed the composition and level of lipoproteins, and accelerated atherosclerosis [4, 21], resulting in endothelial dysfunction [22], that was associated with cardiovascular and cerebrovascular events [23–25]. Reduced eGFR increases stroke incidence and the prevalence of severe neurological deficits [25–28] and is a predictive factor of a poor outcome [29–32]. Recent studies revealed that the mechanisms behind this cerebro-renal interaction may be associated with a reduction in the effectiveness of dynamic cerebral autoregulation in AIS patients with a low eGFR [33]. Moreover, a low eGFR itself is also an independent risk factor for carotid atherosclerosis rather than a traditional vascular risk factor [27, 34, 35].

It is well known that the severity of stroke is the most robust determinant of outcomes in AIS patients. Patients with elevated LDL-C have severe neurological deficits in the CKD population. Whether certain lipid components were associated with functional outcome of stroke remains debatable. Putaala J et al. found that decreased HDL levels predicted adverse outcomes 3 months in young adult stroke patients [11]. In a meta-analysis with 4119 patients, TG levels were linked to stroke mortality [8], whereas a prospective study identified that there were no obvious associations between serum lipids and outcomes [15]. A multicenter clinical trial including 3348 adult patients suggested that AIS patients with high LDL-C (≥ 4.14 mmol/L) and a low eGFR calculated by CysC were independently associated with a poor 1-year outcome [36]. Our study indicated that serum lipids were not independently associated with early functional outcome regardless of eGFR status.

Our findings provide evidence for risk stratification among individuals with CKD and underscore the clinical significance of LDL-C levels as a lipid indicator of stroke severity in patients with CKD. Intensive statin treatment could effectively reduce LDL-C level and the risk of stroke in patients with CKD [37]. A large cohort prospective study confirmed that lowing LDL-C was associated with decreased stroke severity and improved functional outcomes in AIS patients [38]. It also indirectly indicated the effect of LDL-C on stroke severity. Given the detriment of elevated LDL levels in AIS patients, it may be beneficial to control LDL-C levels more strictly in patients with CKD than in those without CKD.

Some limitations should be taken into account when interpreting the results. Firstly, the retrospective observational study could not identify a definite causal relationship between LDL-C levels and stroke severity in CKD patients. Secondly, the single-center data may have exhibited the regional bias, although extensively adjusting for possible potential confounders. Thirdly, we only followed up until discharge, which may not be long enough to observe the effect of lipid profiles on outcome of AIS patients with and without CKD. There is a need for multicenter large-scale prospective studies to confirm whether a lower LDL goal is more beneficial to the long-term outcomes in CKD patients than in the general population to provide evidence for different therapeutic targets of lipids among patients with renal impairment in risk-stratified management. Notwithstanding these limitations, this is the first study of interaction and independent associations of lipid components with severity and outcome of stroke according to lipid level quartiles by CKD stratification. In addition, the eGFR was based on the CKD-EPI equation for Asians, which is considered more precise than the Modification of Diet in Renal Disease Study equation [20].

## Conclusions

We present evidence on the association between elevated LDL-C levels and severe neurological deficits in AIS patients with CKD but not among those without CKD. The mechanism of pathophysiological interaction deserves intensive study.
